# Repeat multiplex PCR gastrointestinal panel testing within 14 days yields minimal additional diagnostic information: a multicenter cohort study

**DOI:** 10.1017/ice.2026.10464

**Published:** 2026-07

**Authors:** Jeffrey Shu, Hannah Wang, Anisha Misra, Daniel D. Rhoads, Amy S. Nowacki, Jarrod E. Dalton, Abhishek Deshpande

**Affiliations:** 1 Cleveland Clinic Lerner College of Medicine of Case Western Reserve University, USA; 2 Cleveland Clinic Main Campus Hospital: Cleveland Clinic, USA; 3 Cleveland Clinic’s Robert J Tomsich Pathology and Laboratory Medicine Institute, USA; 4 Cleveland Clinic Health System: Cleveland Clinic, USA; 5 Alice L. Walton School of Medicine, Bentonville, USA

## Abstract

**Introduction:**

Expanded multiplex PCR gastrointestinal panels (GIPs) are routinely ordered to diagnose infectious diarrhea. However, recommendations for repeat GIP testing are limited, resulting in variable testing practices. We evaluated the diagnostic yield of repeat GIP testing within 14 days.

**Methods:**

We conducted a retrospective cohort study of adults (age ≥ 18 years) tested with GIPs across 12 hospitals and outpatient centers (2019–2024). We analyzed the first diarrheal episode per patient, excluding cases with invalid/missing results and *C. difficile* results. Repeat testing was defined as a GIP completed within 14 days of an index GIP, excluding confirmatory testing using the same stool sample. The primary outcome was diagnostic yield (new pathogen detection), and the secondary outcome was pathogen persistence (same pathogen detection).

**Results:**

Among 16,502 patients, 507 (3.1%) underwent repeat GIP testing within 14 days (median interval: 6.3 days; IQR: 2.7–9.8). Only 4.6% [19/415] index-negative patients and 2.2% [2/92] index-positive patients detected new pathogens on repeat testing, with 51% [47/92] index-positive patients demonstrating persistence of at least one pathogen from their initial test. The number needed to test (NNT) to identify one new pathogen was 24 (95% CI: 16–39) tests overall, and 127 (95% CI: 50–455) tests to identify one new pathogen warranting antimicrobial treatment. Most repeat testing (86% [436/507]) was ordered by a different clinician.

**Discussion:**

Repeat GIP testing within 14 days rarely provided new diagnostic information, highlighting the limited utility of early repeat testing. Institutional policies discouraging repeat GIPs within 14 days may improve diagnostic stewardship.

## Introduction

Expanded multiplex gastrointestinal panels (GIPs), simultaneously testing over 20 pathogens, are now routinely used for rapid and comprehensive diagnosis of infectious diarrhea.^1–3^ However, clinical interpretation of GIP results may be difficult due to the nature of molecular diagnostics.^4,5^ Their high sensitivity may lead to the detection of persistent organisms with unclear clinical significance and may not adequately differentiate between viable pathogenic organisms and remnant nucleic acids or non-pathogenic colonizers.^6–9^

Clinicians may order repeat testing for a variety of reasons: to confirm potential false positives, evaluate new or progressing symptoms, confirm pathogen clearance (“test-of-cure”), or fulfill public health reporting for outbreak-prone organisms.^7,8,10^ Multiple clinical society guidelines have cautioned against repeat stool testing for *Clostridioides difficile* within 7 days, citing low yield and high false-positive rates.^4,11,12^ However, similar guidance for GIPs is lacking, leading to variability in testing practices and a gap in knowledge about the clinical utility of early repeat testing. One single-center study of 106 patients with repeat GIP testing demonstrated that follow-up testing within four weeks rarely led to a change in results.^10^ Another single-center study of 228 patients identified a repeat testing rate of 1.8% within four weeks.^13^ However, these studies were limited by small sample sizes and may not accurately identify differences between repeat tests across a large 4-week testing interval.

The objectives of our study were to 1) characterize patients receiving repeat GIP testing within 14 days of an index GIP, 2) evaluate the diagnostic yield of repeat testing, 3) evaluate pathogen persistence on repeat testing, and 4) identify which clinical settings contributed most to repeat testing. We hypothesized that repeat testing within 14 days would rarely detect new pathogens and that persistent positivity would be common among initially positive patients. Our study aims to inform evidence-based policies around GIP utilization and diagnostic stewardship.

## Methods

### Study population and data extraction

We conducted a multicenter retrospective cohort study including adults (≥18 years) tested with a GIP within an integrated health system from January 1, 2019, through March 1, 2024. This healthcare system comprised 12 hospitals and associated outpatient centers across Ohio and Florida. GIPs were defined as completed orders for the BioFire FilmArray Gastrointestinal Panel® (bioMérieux, France). Demographics, testing location, sample collection date, comorbidities, and microbiology results were extracted from electronic health records. High-risk patients were defined as patients with ≥1 of 10 predefined chronic conditions identified through International Classification of Diseases (ICD-10) codes documented prior to or at the time of testing, as previously described^14^ (Supplementary Table S1). Low-risk patients were those with none of these 10 predefined chronic conditions. Comparative epidemiology of diarrheal organisms detected by GIP in this cohort, as well as overall GIP yield, were previously described by Shu *et al*.^14^ Encounter setting was identified by the department ordering the GIP and classified as inpatient, outpatient, or emergency department. Ordering providers were identified by National Provider Identifier (NPI) and provider specialties were extracted from National Plan and Provider Enumeration System (NPPES) databases.^15^ Manual chart review of 50 randomly selected patients was conducted to validate data accuracy.

### Laboratory testing practices

The BioFire FilmArray GIP simultaneously tests for 21 pathogens (12 bacteria, 5 viruses, 4 parasites).^1^ We analyzed the first diarrheal case (test interval spanning 14 calendar days) per patient, excluding cases with invalid/missing results and *Clostridioides difficile* results. *C. difficile* results on the GIP were excluded from analysis due to variations in laboratory reporting and preexisting guidelines discouraging *C. difficile* repeat testing. Repeat testing was defined as a second GIP completed within 14 days of an index GIP (calendar days between sample collection), excluding confirmatory testing conducted on the same stool sample. For patients with multiple repeat tests, only the test with the largest testing interval within 14 days was analyzed. GIP testing was ordered at the discretion of treating clinicians, with no institutional policies or restrictions in place. Institutional best practices encourage providers to reserve GIP usage for individuals meeting criteria for testing per IDSA recommendations.^5^ All GIP tests were processed using standard laboratory protocols.^16^ Following the January 2024 recall due to norovirus concerns, all norovirus positives were repeat tested on the same platform and only reported as positive if reproducibly positive.^17^ These repeat tests done for quality control were not considered a repeat GIP test.

### Outcomes and definitions

Positivity was defined as detection of any pathogen on the GIP and all analyses were stratified by results of the index GIP (index negative vs index positive). The primary outcome was diagnostic yield of repeat GIP testing, defined as detection of any new pathogen not detected on the index GIP. The secondary outcome was pathogen persistence, defined as detection of the same organism on both index and repeat testing, among index-positive patients. Antibiotic-treatable organisms were previously defined as bacteria and parasites with guideline-recommended antimicrobial therapy per Infectious Diseases Society of America (IDSA) guidelines: *Campylobacter spp.*, *Plesiomonas shigelloides, Salmonella spp.*, *Shigella*/EIEC*; Vibrio spp.* (*parahaemolyticus, vulnificus*), *Vibrio cholerae*, *Yersinia enterocolitica*, *Cyclospora cayetanensis*, *Entamoeba histolytica*, *and Giardia lamblia*.^5,18^

### Statistical analysis

Baseline characteristics were presented as median and interquartile range (IQR) for continuous variables and proportions (%) for categorical variables within each testing group (repeat testing vs single testing only). Pearson’s χ^2^ tests and Wilcoxon rank-sum tests compared baseline characteristics for categorical and continuous variables across testing groups, respectively. We calculated 95% confidence intervals (95% CI) for proportions using the Clopper-Pearson method for primary and secondary outcomes. Number needed to test (NNT) was calculated as the inverse of the diagnostic yield for any organism and organisms warranting antibiotic treatment, with 95% CIs derived from the corresponding confidence limits. Multiple logistic regression analyzed the associations between testing interval and diagnostic yield, controlling for index-result. Heatmap visualization was used to display changes in pathogen detection patterns between index and repeat testing. Care transition patterns were visualized using Sankey flow diagrams to illustrate patient movement between clinical settings. Provider continuity was assessed by matching NPI codes or ordering departments between index and repeat test orders. Analyses were conducted with R version 4.5.1 on a complete-case basis and utilized a 5% significance level. Given the exploratory nature of secondary and subgroup analyses, no adjustments were made for multiple comparisons.

### Sensitivity analyses

Sensitivity analyses were performed using 7-, 14-, 28-, and 56-day testing intervals to evaluate the effect of the repeat-testing window on the primary and secondary outcomes. We used a Pearson’s χ^2^ tests to compare differences in diagnostic yield of repeat testing between “high-risk” and “low-risk” patients, as previously described.^14^ Subgroup analyses were conducted on 1) patients receiving repeat testing with an index test between January 26, 2024 through March 1, 2024, and 2) all repeat testing excluding norovirus results to evaluate the impact of the 2024 BioFire FilmArray GIP recall related to concerns about norovirus false positivity.^17^

## Results

### Baseline characteristics of patients receiving repeat testing

During the study period, 16,502 adult patients underwent GIP testing across 12 hospitals, with an overall detection rate of 22%. Of these, 507 patients (3.1%, 95% CI: 2.8–3.4%) received repeat testing within 14 days of their index test, with a median interval of 6.2 days (IQR: 2.6–9.7 days) between tests.

Of the 507 repeat tests, 415 (82%) were conducted in patients with index-negative results compared to 92 (18%) with index-positive results (Table 1). Among the 92 index-positive patients, 16 (17%) identified multiple pathogens, detecting either two or three organisms. Patients who underwent repeat testing differed significantly from those with single testing across multiple baseline characteristics. Patients with repeat testing were older (median age 69 vs 66 years; *P* = .003), more likely to be male (46% vs 40%; *P* = .019), and more likely to have immunocompromising conditions including organ transplant (7.7% vs 3.0%; *P* < .001), inflammatory bowel disease (9.1% vs 6.1%; *P* = .005), metastatic cancer (9.1% vs 5.7%; *P* = .001), and primary immunodeficiency (7.9% vs 5.1%; *P* = .004).

**Table 1.** Baseline characteristics of patients receiving repeat tests compared to single testing only Table 1 long description.

Characteristic	Single test, N = 15,995	Repeat test, N = 507	*P*-value
**Sex (Male), N (%)**	6,458 (40)	231 (46)	.019
**Age, median (IQR), years**	66 (51, 77)	69 (56, 78)	.003
**Race (White), N (%)**	12,814 (80)	428 (84)	.047
**High-risk conditions, N (%)**			
Rheumatologic disorder	913 (5.7)	32 (6.3)	.6
Lymphoma	241 (1.5)	6 (1.2)	.6
Leukemia	246 (1.5)	10 (2.0)	.4
Metastatic cancer	912 (5.7)	46 (9.1)	.001
Transplant recipient	484 (3.0)	39 (7.7)	<.001
HIV	151 (0.9)	5 (1.0)	.8
Inflammatory bowel disease	969 (6.1)	46 (9.1)	.005
Irritable bowel syndrome	1,071 (6.7)	39 (7.7)	.4
Primary immunodeficiency	809 (5.1)	40 (7.9)	.004
**Initial result, N (%)**			
Index negative	–	415 (82)	–
Index positive	–	92 (18)	–
**Initial test positive, N (%)**	3,491 (22)	92 (18)	.048
**Year, N (%)**	–	–	.3
2019	1,658 (10)	47 (9.3)	–
2020	2,392 (15)	72 (14)	–
2021	2,976 (19)	84 (17)	–
2022	3,395 (21)	109 (21)	–
2023	4,593 (29)	168 (33)	–
2024 (partial)	981 (6.1)	27 (5.3)	–
**Season, N (%)**	–	–	.5
Fall	3,697 (23)	108 (21)	–
Spring	3,760 (24)	132 (26)	–
Summer	3,964 (25)	120 (24)	–
Winter	4,574 (29)	147 (29)	–
**Location, N (%)**	–	–	.003
Florida	7,835 (49)	282 (56)	–
Ohio	8,160 (51)	225 (44)	–
**Initial test setting, N (%)**	–	–	<.001
Emergency department	3,173 (20)	115 (23)	–
Inpatient	9,927 (62)	341 (67)	–
Outpatient	2,895 (18)	51 (10)	–
**Testing interval, median (IQR), days**	–	6.2 (2.6, 9.7)	–

**Figure 1.**
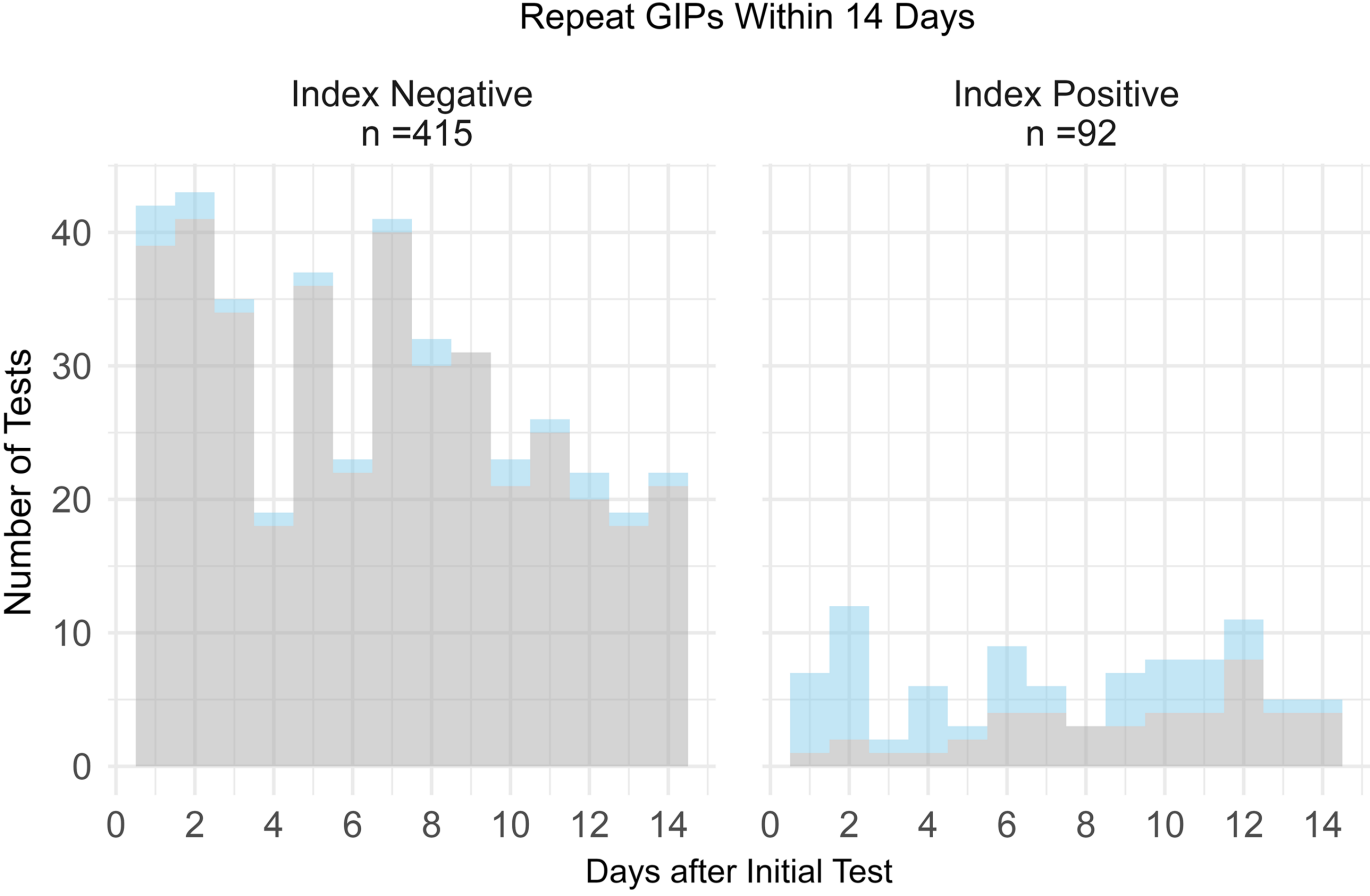
Positivity of repeat GIPs within 14 days, stratified by index GIP result. Each bar represents the total number of repeat GIP tests ordered per day after index test. Light blue represents positive repeat GIPs, light gray represents negative repeat GIPs.

Index-negative repeat GIPs became positive in 19/415 cases (4.6%, 95% CI: 2.8–7.1%) and index-positive repeat GIPs remained positive in 47/92 cases (51%, 95% CI: 40–62%) (Figure 1).

### Primary outcome: diagnostic yield of repeat testing

**Figure 2.**
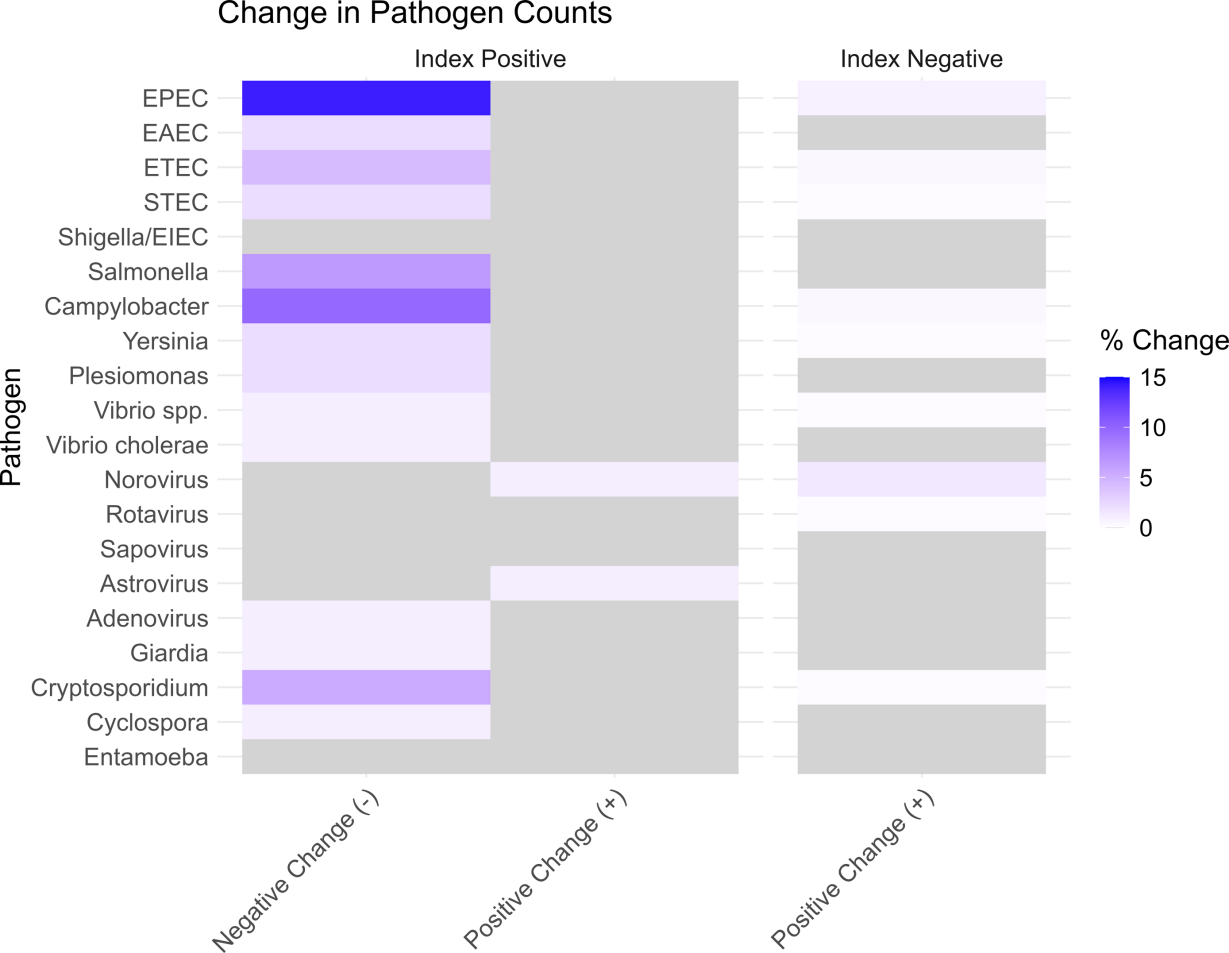
Figure 2 long description. Heatmap of changes in pathogen detection on repeat testing, stratified by index GIP result. Light gray represents no change (0%). Abbreviations: EPEC, enteropathogenic *E. coli*; EAEC, enteroaggregative *E. coli*; ETEC, enterotoxigenic *E. coli*; STEC, Shiga toxin-producing *E. coli*; EIEC, enteroinvasive *E.coli*.

The overall diagnostic yield of repeat GIP testing was 4.1% (95% CI: 2.6–6.3%) representing 21 of 507 repeat tests detecting new pathogens (Figure 2). Diagnostic yield was not significantly different across testing intervals: ≤3 days (reference), 4–7 days (OR 0.65, 95% CI: 0.16–2.32), and 8–14 days (OR 1.21; 95% CI: 0.45–3.58) (*P* = .6) or between index-negative (reference) and index-positive tests (OR 0.45, 95% CI: 0.01–1.58, *P* = .2).

Among patients with index-negative tests, 19 of 415 (4.6%; 95% CI: 2.8–7.1%) detected new pathogens on repeat testing. The most frequently identified organisms in these patients were norovirus (n = 7), enteropathogenic *Escherichia coli* (EPEC) (n = 4), and *Campylobacter* species (n = 2). Conversely, only 2 of 92 index-positive tests (2.2%; 95% CI: 0.3%–7.7%) detected new pathogens on repeat testing. Both index-positive cases involved new detection of viral pathogens (one case each of norovirus and astrovirus) in patients readmitted with persistent diarrheal symptoms after discharge.

Of the 21 new pathogens detected on repeat testing, only 4 new detections (19%) involved organisms with guideline-recommended antimicrobial treatment: *Campylobacter* (n = 2), *Yersinia spp.* (n = 1), and *Vibrio spp.* (n = 1). (Supplementary Table S2). The NNT to identify one new pathogen was 24 (95% CI: 16–39) tests overall, and 127 (95% CI: 50–455) tests to identify one new pathogen warranting antimicrobial treatment.

### Secondary outcome: pathogen persistence

Another common reason that clinicians may repeat GIP testing is to assess pathogen clearance for test-of-cure or for public health reporting purposes. Of 92 index-positive tests, GIPs were repeated for those with bacterial detections in 63% of cases, viruses in 37%, and parasites in 12%. Among these index-positive patients, 47 (51%; 95% CI: 40.4%–61.7%) demonstrated persistence of at least one pathogen from their initial test (Figure 2). Persistence rates were similar among viral (n = 20/34; 58.8%), bacterial (n = 27/58; 46.6%) and parasitic pathogens (n = 4/11, 36.4%) (*P* = .28), though interpretation is limited due to small sample sizes. The most prevalent persistent organisms were norovirus (n = 13/27, 48.1%), EPEC (9/22, 40.9%), and *Campylobacter* (n = 7/16, 43.8%).

Complete pathogen clearance (“negative change”) occurred in 45/92 (49%; 95% CI: 38.6–59.1%) index-positive patients when GIPs were repeated within 14 days. The most cleared organisms were norovirus (n = 14/27, 51.9%), EPEC (n = 13/22, 59.1%), *Campylobacter* (n = 9/16, 56.3%), *Salmonella* (n = 6/9, 66.7%), and *Cryptosporidium* (n = 5/8, 62.5%). Patients with complete pathogen clearance (median 9.2 days, IQR: 6.6–11.3) repeated their test in a longer test interval compared to those without pathogen clearance (median 5.0 days, IQR: 1.4–9.6) (*P* < .001).

### Healthcare utilization and care transitions

**Figure 3.**
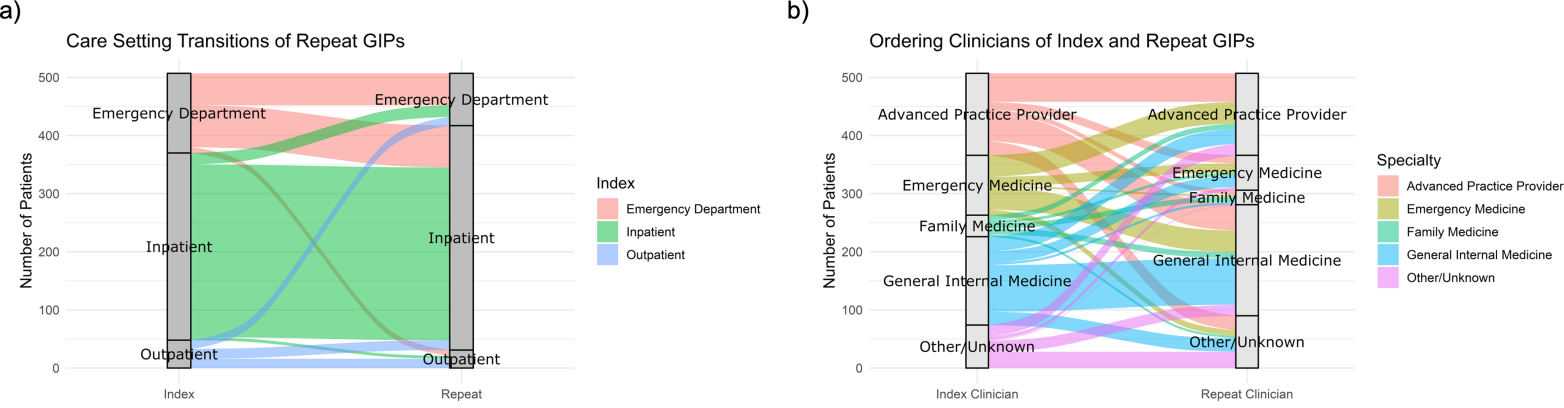
Flow diagrams of transitions between index and repeat GIP tests for a) clinical setting and b) clinician specialty.

To identify system factors that impact repeat testing, we investigated GIP repeat ordering factors such as encounter setting, clinician specialty, and individual clinician. Most repeat testing occurred during transitions of care (Figure 3). Among the 507 patients receiving repeat tests, 137 (27.0%; 95% CI: 23.2–31.1%**)** had testing performed in a different encounter setting compared to their index test, 325 (64.1%; 95% CI: 59.8–68.3%) by a different clinician specialty, and 436 (86.0%; 95% CI: 82.8–88.6%) by a different individual clinician.

Most repeat testing occurred in inpatient-to-inpatient settings (n = 297) and emergency department to emergency department (n = 55). The most common encounter transitions were from emergency department to inpatient settings (n = 74). There was no significant difference in diagnostic yield in patients with repeat testing conducted in the same encounter setting compared to different encounter settings (3.8% vs 5.0%, *P* = .5).

Only 71 of 507 patients (14.0%) had both tests ordered by the same clinician. Among cases with provider continuity, the diagnostic yield was not significantly different from cases with different ordering providers (2.8% vs 4.4%, *P* = .8) (Table 2).

**Table 2.** Test characteristics of patients receiving repeat testing ordered by the same clinician or different clinicians

Characteristic	Overall, N = 507	Same clinician, N = 71	Different clinician, N = 436
Specialty transition			
Same specialty	182 (36%)	71% (100%)	111 (25%)
Different specialty	325 (64%)	0 (0%)	325 (75%)
Repeat test result			
Positive	66 (13%)	7 (9.9%)	59 (14%)
Negative	441 (87%)	64 (90%)	377 (86%)
Initial test result			
Index negative	415 (82%)	55 (77%)	360 (83%)
Index positive	92 (18%)	16 (23%)	76 (17%)
Detection of new pathogen	21 (4.1%)	2 (2.8%)	19 (4.4%)
Test setting of repeat test			
Emergency department	90 (18%)	4 (5.6%)	86 (20%)
Inpatient	386 (76%)	53 (75%)	333 (76%)
Outpatient	31 (6.1%)	14 (20%)	17 (3.9%)
Testing interval, median (IQR), days	6.2 (2.6, 9.7)	6.3 (2.1, 9.9)	6.2 (2.7, 9.7)

### Sensitivity analyses

Varying the repeat testing window demonstrated consistently low diagnostic yield. Compared to an overall diagnostic yield of GIPs of 22% in our cohort, a 7-day repeat testing window yielded 3.6% new pathogen detection, 14-day window yielded 4.1%, 28-day window yielded 4.9%, and 56-day window yielded 5.5%. (Supplementary Table S3) There was also no significant difference in diagnostic yield in repeat testing between high-risk and low-risk patients (4.1% vs 4.1%; *P* > .9).^14^

In January 2024, a recall was announced related to concerns about norovirus false positives.^17^ To assess the effect of the recall on repeat ordering rates, we conducted subgroup analyses 1) identifying patients with repeat testing following the recall, and 2) reanalyzing the NNT to detect an organism (excluding norovirus results) given concerns about false positivity potential. Between January 26, 2024 and March 1, 2024, 614 patients underwent GIP testing, of which 53 (8.6%) were positive for norovirus. 12/614 (2.0%) patients received a repeat test, including 10 who were index-negative and 2 who were index-positive (1 detecting norovirus and *Campylobacter*, 1 detecting *Campylobacter*). When excluding norovirus results from the overall cohort, the NNT to identify one new pathogen (excluding norovirus) was 37 (95% CI: 22–64) tests overall. There were no changes to the NNT to identify one pathogen warranting antibiotic treatment.

## Discussion

In this multicenter cohort study, we found that repeat GIP testing within 14 days rarely yields new diagnostic information. Over 80% of repeat testing was conducted in patients with index-negative tests. Fewer than 5% of patients identified a new pathogen on repeat testing, with over 120 repeat tests needed to detect one new pathogen warranting antimicrobial treatment. Among index-positive patients, over half continued to test positive for the same pathogen on follow-up. Lastly, most repeat tests were ordered by a different clinician, highlighting that most repeat testing occurs during transitions of care.

The low detection of new pathogens supports that most diarrheal illnesses either resolve spontaneously or do not evolve into new infections within a 14-day period.^5,10,19^ The most common new pathogens (norovirus and EPEC) are self-limiting infections in immunocompetent hosts and do not necessitate antimicrobial therapy.^5^ Additionally, the persistent detection of pathogens on repeat testing in index-positive individuals supports existing concerns about the detection of nonviable organisms in molecular testing, suggesting test-of-cure or public health reporting should not be done with molecular testing.^4,6,19–21^ Culture-independent methods for test-of-cure could also lead to missed detection of difficult-to-treat and multidrug-resistant (MDR) organisms. Culture-based methods remain critical for pathogen characterization and public health reporting in these cases, as it allows for higher specificity and subsequent susceptibility testing.^5,22^ Our findings expand upon a prior smaller single-center study of 106 patients that demonstrates poor clinical utility of repeat GIP testing within 4 weeks and also recommends against using a GIP for test-of-cure purposes.^10^

These findings have implications for diagnostic stewardship. In the absence of new symptoms, repeat GIP testing within two weeks offers minimal added value. Repeat testing was more frequently ordered in index-negative patients, as well as high-risk, immunocompromised patients with no significant difference in diagnostic yield compared to low-risk patients. We observed significant regional variation in repeat testing, with higher rates at Florida sites compared to Ohio, potentially reflecting differences in testing culture, patient populations, or provider training. This highlights the variability in testing practices in the absence of standardized stool testing algorithms and further supports the need for institutional policies or clinical decision support (CDS) tools.

Our finding that most repeat testing was requested by a different clinician has not been previously described in the literature. This could be due to lack of recognition of prior results, the desire to use a routine testing approach regardless of previous tests, or belief that tests-of-cure are required for these pathogens. The risks of repeat testing can include increased costs and overutilization on the healthcare system, unnecessary antimicrobial use for incidental findings, or extended isolation precautions due to persistent detection of nonviable pathogens.^5,23,24^ Future studies should investigate the impact of repeat testing on treatment decisions and healthcare costs. As repeat testing occurs primarily during transitions of care, institutions and patients may benefit from implementing CDS tools (e.g. alerts informing providers of existing prior results) or order restrictions (e.g. hard stops) that discourage repeat GIP testing within 14 days, similar to recommendations for *C. difficile* repeat testing.^6,12,13,25,26^ Previous studies on CDS tools have demonstrated varying effectiveness at limiting unnecessary testing, with hard stops more effective than soft stops.^13,27,28^

This study has several limitations. We aimed to provide evidence-based guidance for institutional policies regarding repeat molecular testing, while acknowledging that certain clinical scenarios may warrant exceptions to general recommendations. First, its retrospective nature may be subject to unmeasured confounding. For example, false positive recalls for norovirus, as well as cross-reactivity concerns with *E. coli* species, may affect the specificity of these tests and the organisms identified in our study.^8,17^ However, sensitivity analyses excluding norovirus results and patients tested after the January 2024 norovirus recall did not significantly change the conclusions from this study. Second, we were unable to capture clinical rationale for repeat testing (such as new symptoms, test-of-cure, public health reporting, or unintended repeat testing) due to the absence of standardized clinical documentation. Third, the time line between symptom onset and testing is not standardized. Variations in when a patient is tested during their disease course make it difficult to generalize pathogen persistence rates. Additionally, while our study analyzed repeat testing within 14 days, sensitivity analyses suggested that diagnostic yield did not substantially change even over longer intervals up to 56 days. The “optimal” cutoff for repeat testing intervals will require future studies with larger sample sizes or prospective designs. Fourth, our study focused on molecular detection methods that may not differentiate between nucleic acids and viable organisms. Future studies should investigate the associations between pathogen persistence rates using culture-independent and culture-based methods. Lastly, although we present one of the largest cohorts in repeat GIP testing, our findings may not generalize to other hospital systems with different institutional guidelines and patient populations. For example, GIP ordering may be influenced by the logistics of hospital systems, with different institutional guidelines, patient populations, and hierarchical ordering practices.

In conclusion, repeat GIP testing within 14 days rarely results in the identification of new pathogens and is most ordered during transitions of care. These findings can be used to support institutional policies that discourage repeat testing within 14 days, which would improve diagnostic testing stewardship. Future studies should explore whether CDS interventions to limit repeat GIP testing can improve diagnostic stewardship and patient outcomes.

## Supporting information

10.1017/ice.2026.10464.sm001 Shu et al. supplementary material 1 Shu et al. supplementary material

10.1017/ice.2026.10464.sm002 Shu et al. supplementary material 2 Shu et al. supplementary material

## Data Availability

Individual-level data collected is not publicly available. All data relevant to this study were presented in the article.

## Supplementary material

The supplementary material for this article can be found at https://doi.org/10.1017/ice.2026.10464.

## Figure 2 Long description

Panel A: A horizontal bar graph comparing changes in pathogen counts for index positive results. The horizontal axis represents the percent change, ranging from 0 to 15 percent. The vertical axis lists different pathogens. The color scheme ranges from light purple to dark purple, indicating the magnitude of positive change. Notable pathogens with positive changes include EPEC, Campylobacter, and Cryptosporidium. Panel B: A horizontal bar graph comparing changes in pathogen counts for index negative results. The horizontal axis represents the percent change, ranging from 0 to 15 percent. The vertical axis lists different pathogens. The bars are uniformly light gray, indicating no change in pathogen counts.

Navigate back to Figure 2.

## Table 1 Long description

The table compares baseline characteristics of patients receiving repeat tests to those receiving single testing only. It has 19 rows and 5 columns. The columns are labeled Characteristic, Single test, N = 15,995, Repeat test, N = 507, and P-value. The rows are labeled with various characteristics such as sex, age, race, and high-risk conditions. Each row provides the number and percentage of patients for each characteristic in both the single test and repeat test groups, along with the P-value indicating the significance of the difference between the groups. Notable trends include differences in age, sex, race, and various high-risk conditions between the two groups.

Navigate back to Table 1.
