# Supplementary material for: Repeat multiplex PCR gastrointestinal panel testing within 14 days yields minimal additional diagnostic information: a multicenter cohort study
Source: Infect Control Hosp Epidemiol. 2026 May 15;47(7):703–9. doi: 10.1017/ice.2026.10464 (PMC13315542; doi:10.1017/ice.2026.10464)
Supplement: Shu et al. supplementary material 1 — Shu et al. supplementary material [file S0899823X26104644sup001.docx]

**Supplementary Table S1:** Identification codes for high-risk conditions

| **High-risk Condition** | **ICD10 Code** |
| --- | --- |
| Human Immunodeficiency Virus (HIV) | B20-22, 24, O98.71x, Z21 |
| Primary Immunodeficiency | D80-84, 89 |
| Rheumatologic Disease | M05-06, 31.5, 32-34.9, 35.1, 35.3, 36.0 |
| Metastatic cancer | C77-80.2 |
| Transplant Recipients | T86.00, T86.01, T86.02, T86.03, T86.09, T86.10, T86.11, T86.12, T86.13, T86.19, T86.20, T86.21, T86.22, T86.23, T86.290, T86.298, T86.30, T86.31, T86.32, T86.33, T86.39, T86.40, T86.41, T86.42, T86.43, T86.49, T86.5, T86.810, T86.811, T86.812, T86.818, T86.819, T86.830, T86.831, T86.832, T86.838, T86.839, T86.850, T86.851, T86.852, T86.858, T86.859, T86.890, T86.891, T86.892, T86.898, T86.899, T86.90, T86.91, T86.92, T86.93, T86.99, Z48.21, Z48.22, Z48.23, Z48.24, Z48.280, Z48.288, Z48.290, Z48.298, Z94.0, Z94.1, Z94.2, Z94.3, Z94.4, Z94.5, Z94.6, Z94.7, Z94.81, Z94.82, Z94.83, Z94.84, Z94.89, Z94.9, Z98.85 |
| Inflammatory Bowel Disease (IBD) | K50-51, K52.3 |
| Irritable Bowel Syndrome (IBS) | K58.0-K58.9 |
| Lymphoma | C82-86.6, C88.4, C96, Z85.72, Z85.79, Z85.831 |
| Leukemia | C91-95.99, D45, Z85.6 |
| Age ≥ 65 years | N/A |

**Supplementary Table S2: Epidemiology of index and repeat tests in index-negative and index-positive patients**

| **Characteristic** | **Index Negative (N = 415)** | | **Index Positive (N = 92)** | |
| --- | --- | --- | --- | --- |
|  | Index | Repeat | Index | Repeat |
| EPEC |  | 4 (1.0%) | 22 (24%) | 9 (9.8%) |
| EAEC |  | 0 (0%) | 3 (3.3%) | 1 (1.1%) |
| ETEC |  | 2 (0.5%) | 6 (6.5%) | 2 (2.2%) |
| STEC |  | 1 (0.2%) | 3 (3.3%) | 1 (1.1%) |
| *Shigella*/EIEC |  | 0 (0%) | 2 (2.2%) | 2 (2.2%) |
| *Salmonella* |  | 0 (0%) | 9 (9.8%) | 3 (3.3%) |
| *Campylobacter* |  | 2 (0.5%) | 16 (17%) | 7 (7.6%) |
| *Yersinia* |  | 1 (0.2%) | 2 (2.2%) | 0 (0%) |
| *Plesiomonas* |  | 0 (0%) | 3 (3.3%) | 1 (1.1%) |
| *Vibrio* spp. |  | 1 (0.2%) | 1 (1.1%) | 0 (0%) |
| *Vibrio cholerae* |  | 0 (0%) | 1 (1.1%) | 0 (0%) |
| Norovirus |  | 6 (1.4%) | 27 (29%) | 14 (15%) |
| Rotavirus |  | 1 (0.2%) | 4 (4.3%) | 4 (4.3%) |
| Astrovirus |  | 0 (0%) | 1 (1.1%) | 2 (2.2%) |
| Sapovirus |  | 0 (0%) | 1 (1.1%) | 1 (1.1%) |
| Adenovirus |  | 0 (0%) | 1 (1.1%) | 0 (0%) |
| *Cyclospora* |  | 0 (0%) | 1 (1.1%) | 0 (0%) |
| *Cryptosporidium* |  | 1 (0.2%) | 8 (8.7%) | 3 (3.3%) |
| *Giardia* |  | 0 (0%) | 2 (2.2%) | 1 (1.1%) |
| *Entamoeba* |  | 0 (0%) | 0 (0%) | 0 (0%) |
| Any Pathogen Detected |  | 19 (4.6%) | 92 (100%) | 45 (49%) |
| Any Bacteria |  | 11 (2.7%) | 58 (63%) | 25 (27%) |
| Any Virus |  | 7 (1.7%) | 34 (37%) | 20 (22%) |
| Any Parasite |  | 1 (0.2%) | 11 (12%) | 4 (4.3%) |
| Antibiotic Treatable Pathogen |  | 4 (1.0%) | 34 (37%) | 14 (15%) |
| New Pathogen Detected |  | 19 (4.6%) |  | 2 (2.2%) |
| Testing Interval, Median (IQR), days |  | 6.2 (2.6, 9.3) |  | 7.2 (3.3, 10.7) |

**Supplementary Table S3:** Diagnostic yield of repeat testing across 7-, 14-, 28-, and 56-day intervals

| **Testing Window (days)** | **Initial Result** | **N** | **Negative Change** | **Positive Change** |
| --- | --- | --- | --- | --- |
| 7 | Index Negative | 260 |  | 2.1% |
|  | Index Positive | 48 | 19.8% | 0.0% |
|  | Overall | 308 |  | 1.8% |
| 14 | Index Negative | 415 |  | 2.3% |
|  | Index Positive | 92 | 28.3% | 1.1% |
|  | Overall | 507 |  | 2.1% |
| 28 | Index Negative | 641 |  | 2.7% |
|  | Index Positive | 174 | 32.4% | 1.7% |
|  | Overall | 815 |  | 2.5% |
| 56 | Index Negative | 854 |  | 2.5% |
|  | Index Positive | 245 | 35.3% | 3.5% |
|  | Overall | 1099 |  | 2.7% |
